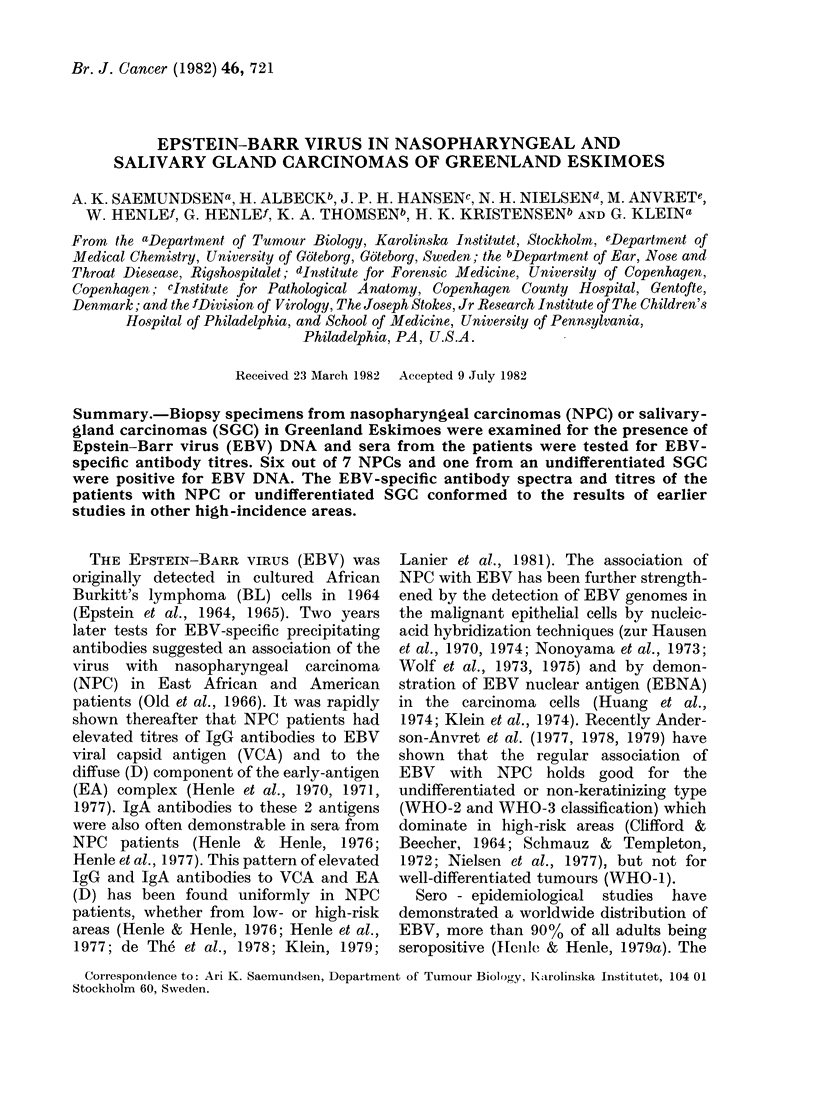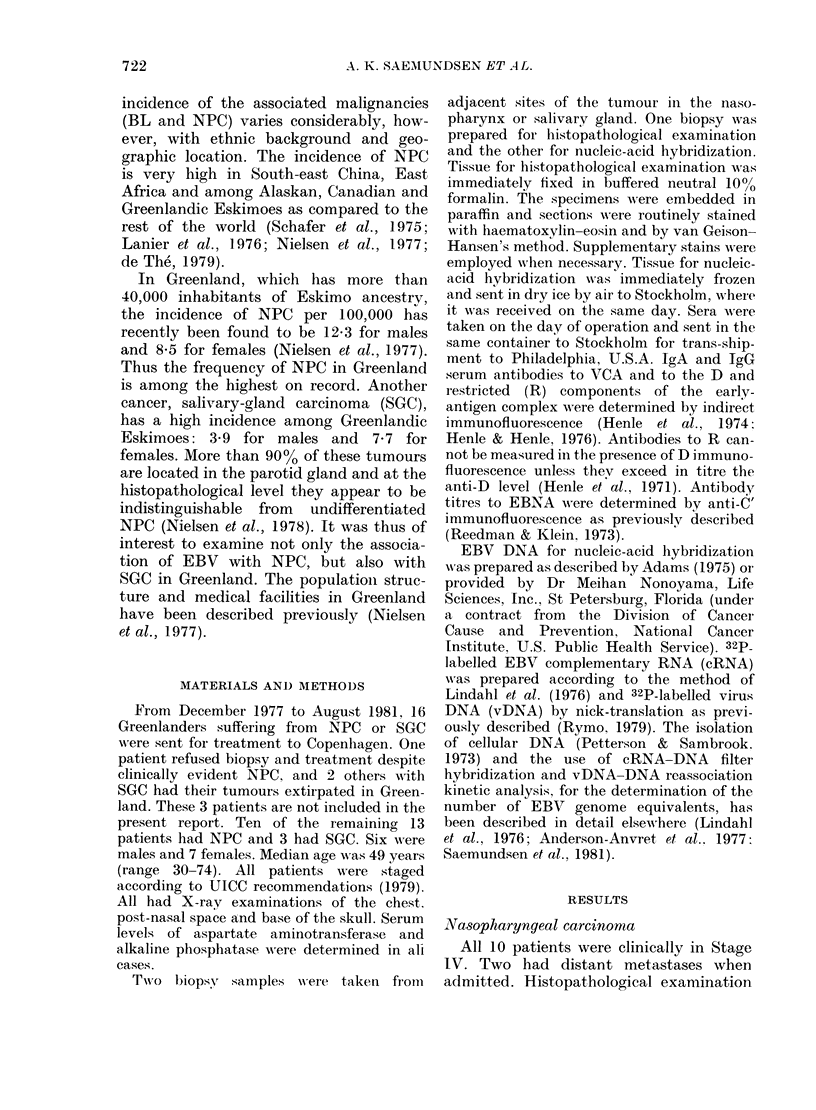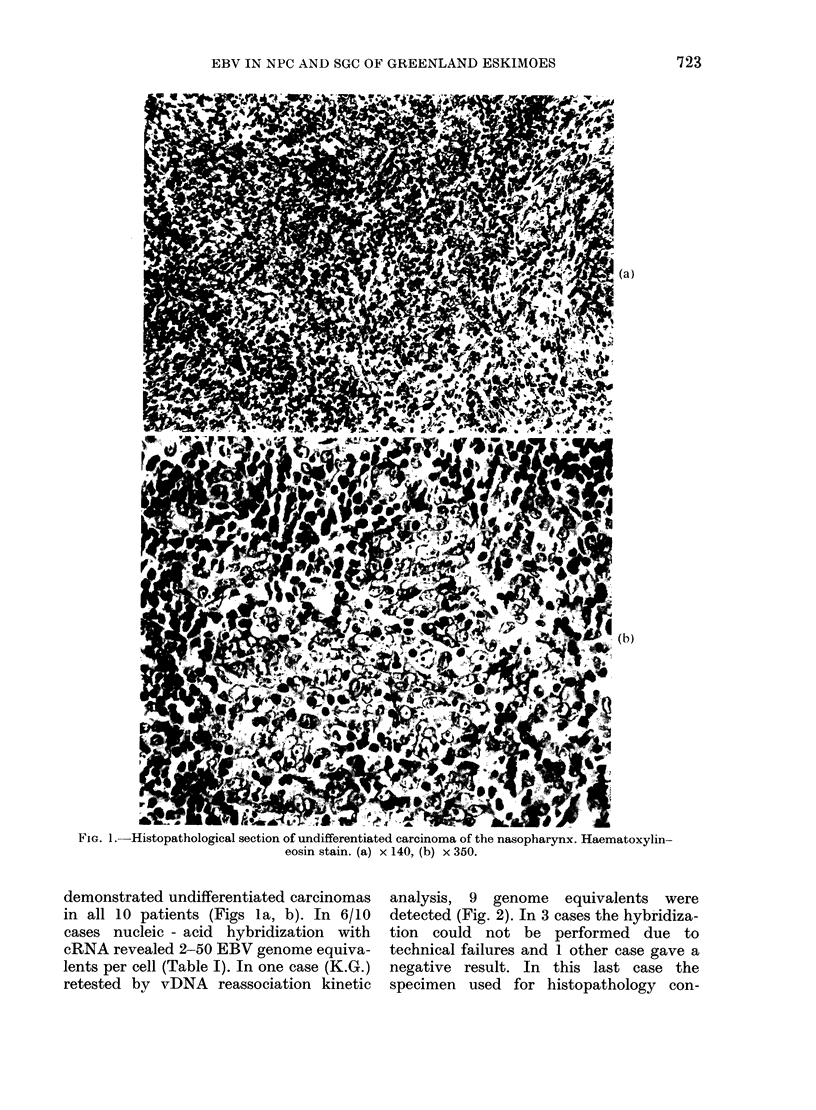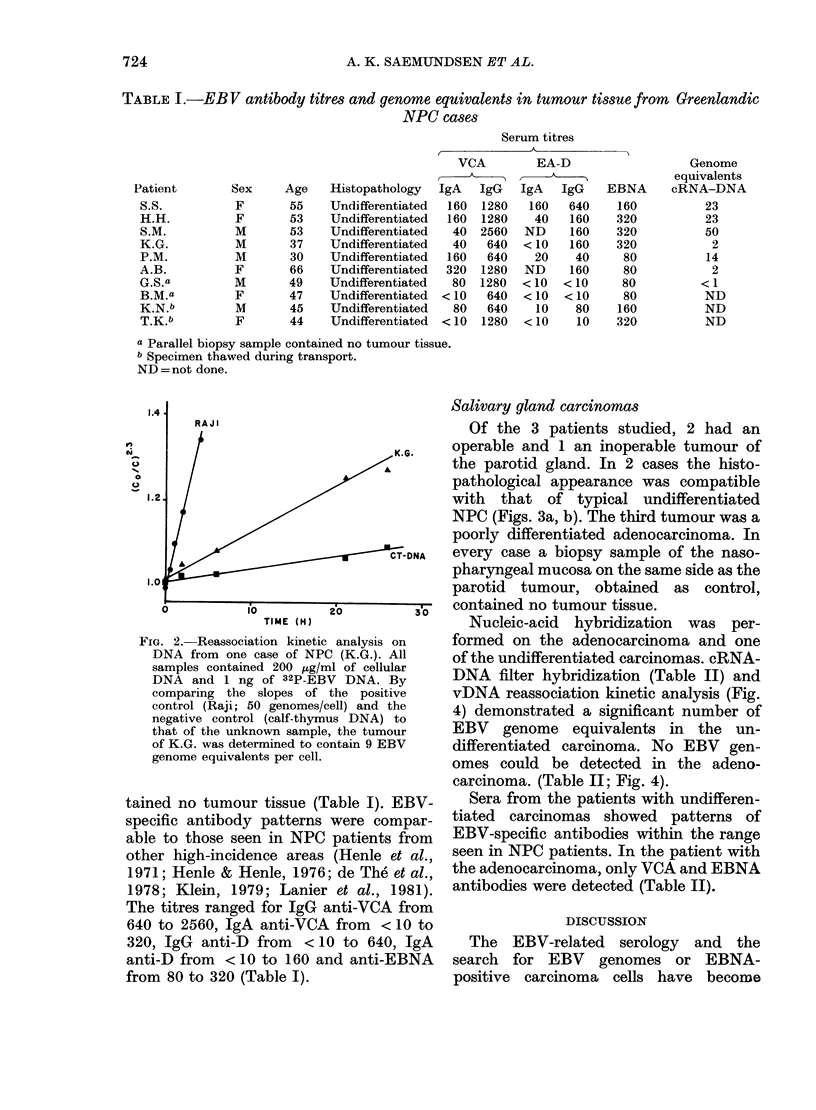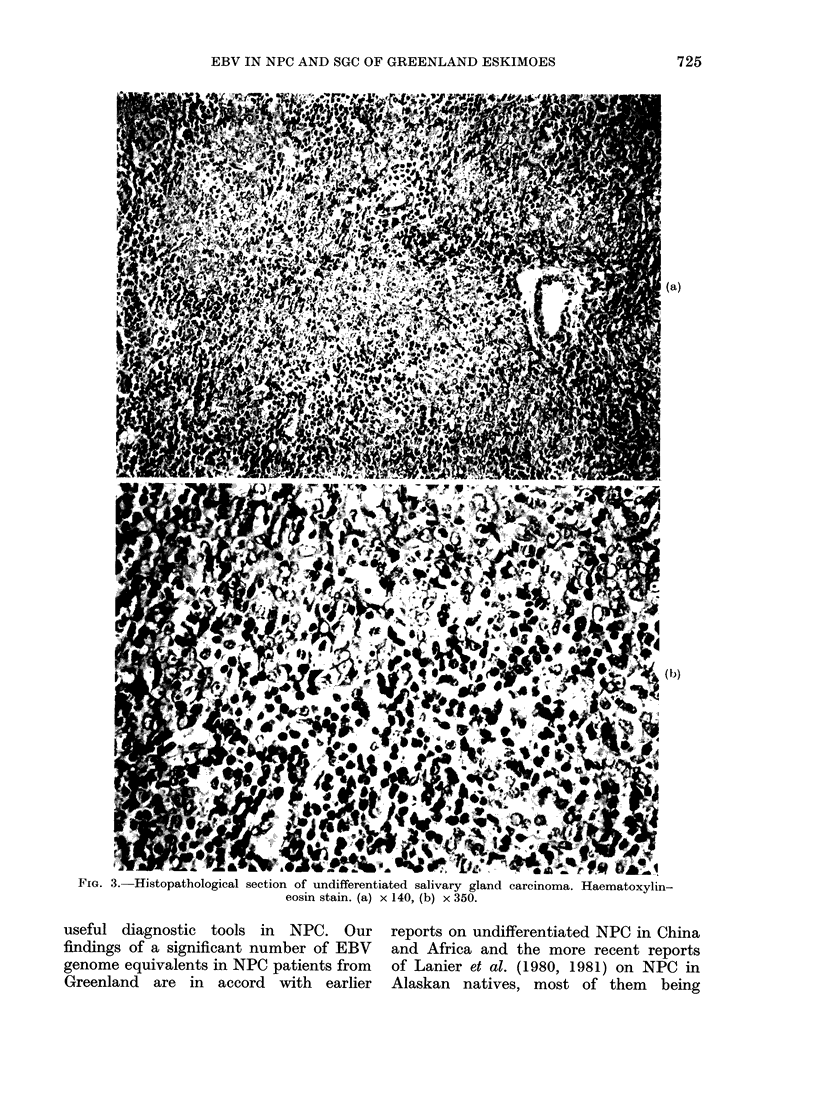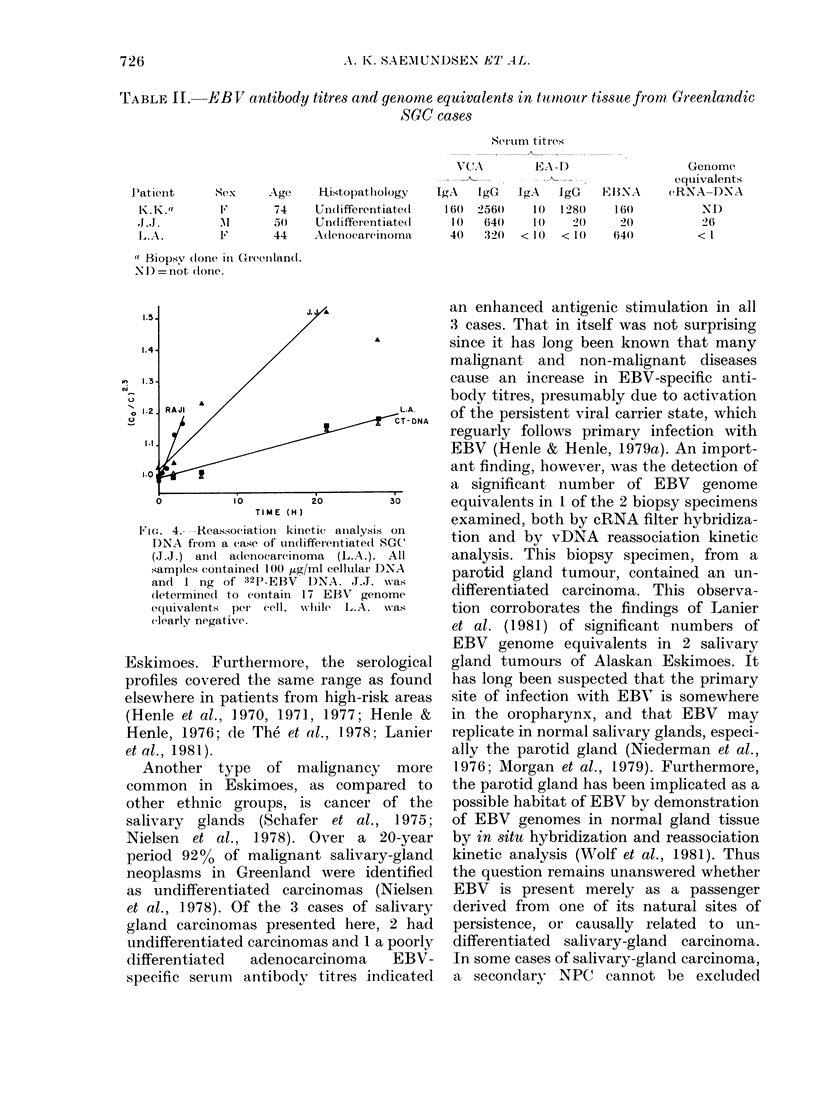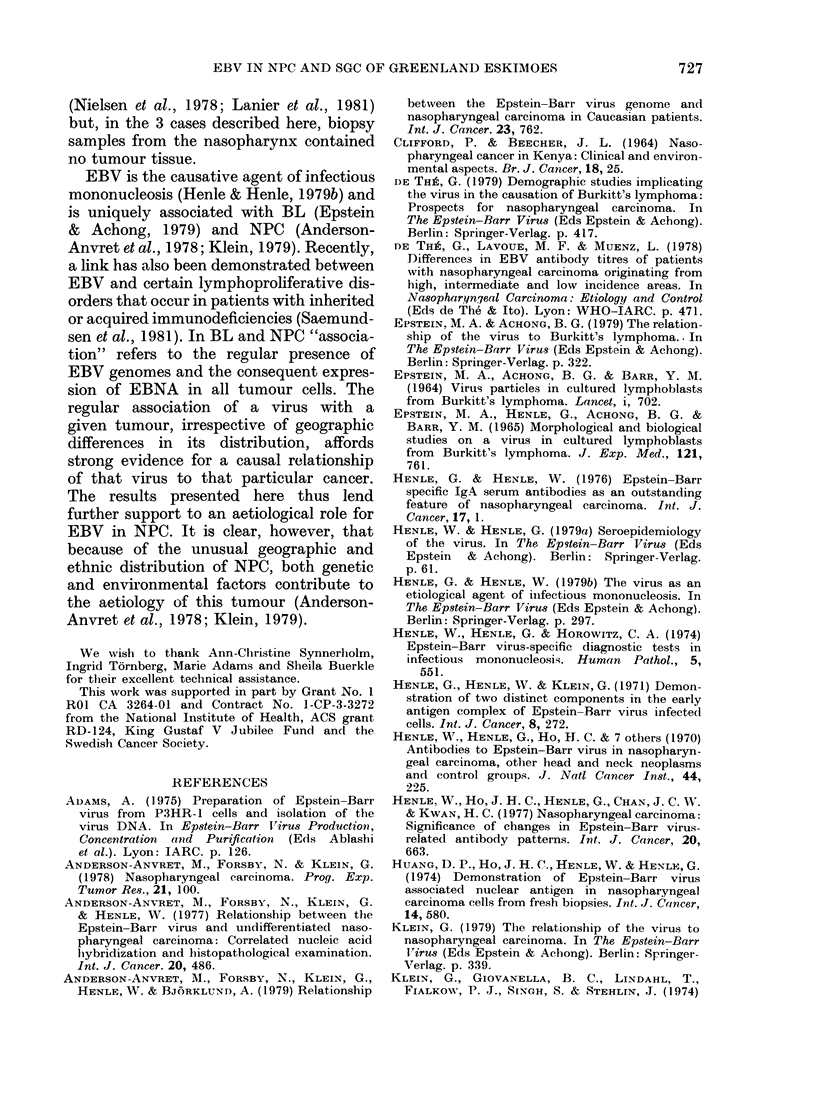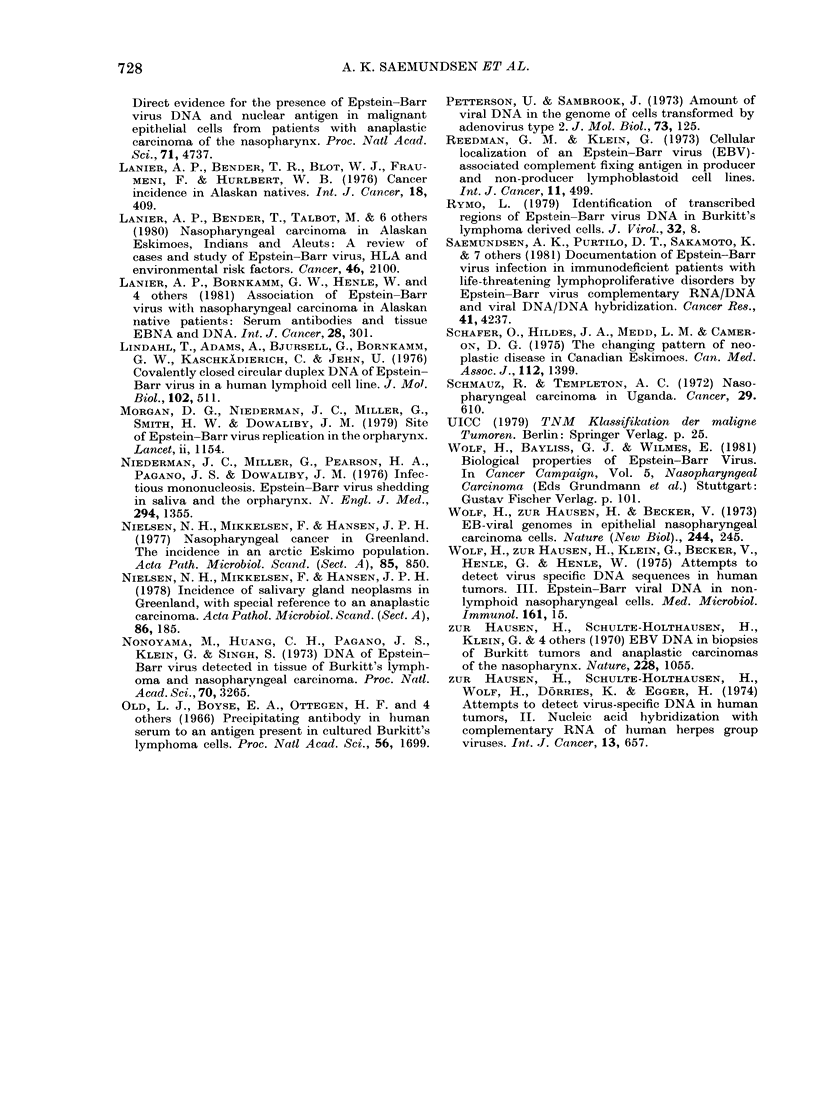# Epstein-Barr virus in nasopharyngeal and salivary gland carcinomas of Greenland Eskimoes.

**DOI:** 10.1038/bjc.1982.264

**Published:** 1982-11

**Authors:** A. K. Saemundsen, H. Albeck, J. P. Hansen, N. H. Nielsen, M. Anvret, W. Henle, G. Henle, K. A. Thomsen, H. K. Kristensen, G. Klein

## Abstract

**Images:**


Br. J. Cancer (1982) 46, 721

### EPSTEIN-BARR VIRUS IN NASOPHARYNGEAL AND SALIVARY GLAND CARCINOMAS OF GREENLAND ESKIMOES

A. K. SAEMUNDSEN[a], H. ALBECK[b], J. P. H. HANSEN[c], N. H. NIELSEN[d], M. ANVRET[e], W. HENLE[f], G. HENLE[f], K. A. THOMSEN[b], H. K. KRISTENSEN[b] AND G. KLEIN[a]

*From the [a]Department of Tumour Biology, Karolinska Institutet, Stockholm, [e]Department of Medical Chemistry, University of Göteborg, Göteborg, Sweden; the [b]Department of Ear, Nose and Throat Diesease, Rigshospitalet; [d]Institute for Forensic Medicine, University of Copenhagen, Copenhagen; [c]Institute for Pathological Anatomy, Copenhagen County Hospital, Gentofte, Denmark; and the [f]Division of Virology, The Joseph Stokes, Jr Research Institute of The Children's Hospital of Philadelphia, and School of Medicine, University of Pennsylvania, Philadelphia, PA, U.S.A.*

Received 23 March 1982 Accepted 9 July 1982

**Summary.—Biopsy specimens from nasopharyngeal carcinomas (NPC) or salivary-gland carcinomas (SGC) in Greenland Eskimoes were examined for the presence of Epstein-Barr virus (EBV) DNA and sera from the patients were tested for EBV-specific antibody titres. Six out of 7 NPCs and one from an undifferentiated SGC were positive for EBV DNA. The EBV-specific antibody spectra and titres of the patients with NPC or undifferentiated SGC conformed to the results of earlier studies in other high-incidence areas.**

THE EPSTEIN-BARR VIRUS (EBV) was originally detected in cultured African Burkitt's lymphoma (BL) cells in 1964 (Epstein *et al.*, 1964, 1965). Two years later tests for EBV-specific precipitating antibodies suggested an association of the virus with nasopharyngeal carcinoma (NPC) in East African and American patients (Old *et al.*, 1966). It was rapidly shown thereafter that NPC patients had elevated titres of IgG antibodies to EBV viral capsid antigen (VCA) and to the diffuse (D) component of the early-antigen (EA) complex (Henle *et al.*, 1970, 1971, 1977). IgA antibodies to these 2 antigens were also often demonstrable in sera from NPC patients (Henle & Henle, 1976; Henle *et al.*, 1977). This pattern of elevated IgG and IgA antibodies to VCA and EA (D) has been found uniformly in NPC patients, whether from low- or high-risk areas (Henle & Henle, 1976; Henle *et al.*, 1977; de Thé *et al.*, 1978; Klein, 1979; Lanier *et al.*, 1981). The association of NPC with EBV has been further strengthened by the detection of EBV genomes in the malignant epithelial cells by nucleic-acid hybridization techniques (zur Hausen *et al.*, 1970, 1974; Nonoyama *et al.*, 1973; Wolf *et al.*, 1973, 1975) and by demonstration of EBV nuclear antigen (EBNA) in the carcinoma cells (Huang *et al.*, 1974; Klein *et al.*, 1974). Recently Anderson-Anvret *et al.* (1977, 1978, 1979) have shown that the regular association of EBV with NPC holds good for the undifferentiated or non-keratinizing type (WHO-2 and WHO-3 classification) which dominate in high-risk areas (Clifford & Beecher, 1964; Schmauz & Templeton, 1972; Nielsen *et al.*, 1977), but not for well-differentiated tumours (WHO-1).

Sero - epidemiological studies have demonstrated a worldwide distribution of EBV, more than 90% of all adults being seropositive (Henle & Henle, 1979*a*). The

Correspondence to: Ari K. Saemundsen, Department of Tumour Biology, Karolinska Institutet, 104 01 Stockholm 60, Sweden.

incidence of the associated malignancies (BL and NPC) varies considerably, however, with ethnic background and geographic location. The incidence of NPC is very high in South-east China, East Africa and among Alaskan, Canadian and Greenlandic Eskimoes as compared to the rest of the world (Schafer *et al.*, 1975; Lanier *et al.*, 1976; Nielsen *et al.*, 1977; de Thé, 1979).

In Greenland, which has more than 40,000 inhabitants of Eskimo ancestry, the incidence of NPC per 100,000 has recently been found to be 12·3 for males and 8·5 for females (Nielsen *et al.*, 1977). Thus the frequency of NPC in Greenland is among the highest on record. Another cancer, salivary-gland carcinoma (SGC), has a high incidence among Greenlandic Eskimoes: 3·9 for males and 7·7 for females. More than 90% of these tumours are located in the parotid gland and at the histopathological level they appear to be indistinguishable from undifferentiated NPC (Nielsen *et al.*, 1978). It was thus of interest to examine not only the association of EBV with NPC, but also with SGC in Greenland. The population structure and medical facilities in Greenland have been described previously (Nielsen *et al.*, 1977).

### MATERIALS AND METHODS

From December 1977 to August 1981, 16 Greenlanders suffering from NPC or SGC were sent for treatment to Copenhagen. One patient refused biopsy and treatment despite clinically evident NPC, and 2 others with SGC had their tumours extirpated in Greenland. These 3 patients are not included in the present report. Ten of the remaining 13 patients had NPC and 3 had SGC. Six were males and 7 females. Median age was 49 years (range 30–74). All patients were staged according to UICC recommendations (1979). All had X-ray examinations of the chest, post-nasal space and base of the skull. Serum levels of aspartate aminotransferase and alkaline phosphatase were determined in all cases.

Two biopsy samples were taken from adjacent sites of the tumour in the nasopharynx or salivary gland. One biopsy was prepared for histopathological examination and the other for nucleic-acid hybridization. Tissue for histopathological examination was immediately fixed in buffered neutral 10% formalin. The specimens were embedded in paraffin and sections were routinely stained with haematoxylin–eosin and by van Geison–Hansen's method. Supplementary stains were employed when necessary. Tissue for nucleic-acid hybridization was immediately frozen and sent in dry ice by air to Stockholm, where it was received on the same day. Sera were taken on the day of operation and sent in the same container to Stockholm for trans-shipment to Philadelphia, U.S.A. IgA and IgG serum antibodies to VCA and to the D and restricted (R) components of the early-antigen complex were determined by indirect immunofluorescence (Henle *et al.*, 1974; Henle & Henle, 1976). Antibodies to R cannot be measured in the presence of D immunofluorescence unless they exceed in titre the anti-D level (Henle *et al.*, 1971). Antibody titres to EBNA were determined by anti-C′ immunofluorescence as previously described (Reedman & Klein, 1973).

EBV DNA for nucleic-acid hybridization was prepared as described by Adams (1975) or provided by Dr Meihan Nonoyama, Life Sciences, Inc., St Petersburg, Florida (under a contract from the Division of Cancer Cause and Prevention, National Cancer Institute, U.S. Public Health Service). $^{32}$P-labelled EBV complementary RNA (cRNA) was prepared according to the method of Lindahl *et al.* (1976) and $^{32}$P-labelled virus DNA (vDNA) by nick-translation as previously described (Rymo, 1979). The isolation of cellular DNA (Petterson & Sambrook, 1973) and the use of cRNA–DNA filter hybridization and vDNA–DNA reassociation kinetic analysis, for the determination of the number of EBV genome equivalents, has been described in detail elsewhere (Lindahl *et al.*, 1976; Anderson-Anvret *et al.*, 1977; Saemundsen *et al.*, 1981).

### RESULTS

### *Nasopharyngeal carcinoma*

All 10 patients were clinically in Stage IV. Two had distant metastases when admitted. Histopathological examination

FIG. 1.—Histopathological section of undifferentiated carcinoma of the nasopharynx. Haematoxylin-eosin stain. (a) × 140, (b) × 350.

demonstrated undifferentiated carcinomas in all 10 patients (Figs 1a, b). In 6/10 cases nucleic - acid hybridization with cRNA revealed 2–50 EBV genome equivalents per cell (Table I). In one case (K.G.) retested by vDNA reassociation kinetic analysis, 9 genome equivalents were detected (Fig. 2). In 3 cases the hybridization could not be performed due to technical failures and 1 other case gave a negative result. In this last case the specimen used for histopathology con-

TABLE I.—*EBV antibody titres and genome equivalents in tumour tissue from Greenlandic NPC cases*

| | | | | Serum titres | | | | | |
|---|---|---|---|---|---|---|---|---|---|
| | | | | VCA | | EA-D | | | Genome equivalents |
| Patient | Sex | Age | Histopathology | IgA | IgG | IgA | IgG | EBNA | cRNA–DNA |
| S.S. | F | 55 | Undifferentiated | 160 | 1280 | 160 | 640 | 160 | 23 |
| H.H. | F | 53 | Undifferentiated | 160 | 1280 | 40 | 160 | 320 | 23 |
| S.M. | M | 53 | Undifferentiated | 40 | 2560 | ND | 160 | 320 | 50 |
| K.G. | M | 37 | Undifferentiated | 40 | 640 | < 10 | 160 | 320 | 2 |
| P.M. | M | 30 | Undifferentiated | 160 | 640 | 20 | 40 | 80 | 14 |
| A.B. | F | 66 | Undifferentiated | 320 | 1280 | ND | 160 | 80 | 2 |
| G.S.[a] | M | 49 | Undifferentiated | 80 | 1280 | < 10 | < 10 | 80 | < 1 |
| B.M.[a] | F | 47 | Undifferentiated | < 10 | 640 | < 10 | < 10 | 80 | ND |
| K.N.[b] | M | 45 | Undifferentiated | 80 | 640 | 10 | 80 | 160 | ND |
| T.K.[b] | F | 44 | Undifferentiated | < 10 | 1280 | < 10 | 10 | 320 | ND |

[a] Parallel biopsy sample contained no tumour tissue.
[b] Specimen thawed during transport.
ND = not done.

FIG. 2.—Reassociation kinetic analysis on DNA from one case of NPC (K.G.). All samples contained 200 μg/ml of cellular DNA and 1 ng of $^{32}$P-EBV DNA. By comparing the slopes of the positive control (Raji; 50 genomes/cell) and the negative control (calf-thymus DNA) to that of the unknown sample, the tumour of K.G. was determined to contain 9 EBV genome equivalents per cell.

tained no tumour tissue (Table I). EBV-specific antibody patterns were comparable to those seen in NPC patients from other high-incidence areas (Henle *et al.*, 1971; Henle & Henle, 1976; de Thé *et al.*, 1978; Klein, 1979; Lanier *et al.*, 1981). The titres ranged for IgG anti-VCA from 640 to 2560, IgA anti-VCA from < 10 to 320, IgG anti-D from < 10 to 640, IgA anti-D from < 10 to 160 and anti-EBNA from 80 to 320 (Table I).

*Salivary gland carcinomas*

Of the 3 patients studied, 2 had an operable and 1 an inoperable tumour of the parotid gland. In 2 cases the histopathological appearance was compatible with that of typical undifferentiated NPC (Figs. 3a, b). The third tumour was a poorly differentiated adenocarcinoma. In every case a biopsy sample of the nasopharyngeal mucosa on the same side as the parotid tumour, obtained as control, contained no tumour tissue.

Nucleic-acid hybridization was performed on the adenocarcinoma and one of the undifferentiated carcinomas. cRNA-DNA filter hybridization (Table II) and vDNA reassociation kinetic analysis (Fig. 4) demonstrated a significant number of EBV genome equivalents in the undifferentiated carcinoma. No EBV genomes could be detected in the adenocarcinoma. (Table II; Fig. 4).

Sera from the patients with undifferentiated carcinomas showed patterns of EBV-specific antibodies within the range seen in NPC patients. In the patient with the adenocarcinoma, only VCA and EBNA antibodies were detected (Table II).

### DISCUSSION

The EBV-related serology and the search for EBV genomes or EBNA-positive carcinoma cells have become

(a)
(b)

Fig. 3.—Histopathological section of undifferentiated salivary gland carcinoma. Haematoxylin–eosin stain. (a) × 140, (b) × 350.

useful diagnostic tools in NPC. Our findings of a significant number of EBV genome equivalents in NPC patients from Greenland are in accord with earlier reports on undifferentiated NPC in China and Africa and the more recent reports of Lanier *et al.* (1980, 1981) on NPC in Alaskan natives, most of them being

TABLE II.—*EBV antibody titres and genome equivalents in tumour tissue from Greenlandic SGC cases*

| | | | | Serum titres | | | | | |
|---|---|---|---|---|---|---|---|---|---|
| | | | | VCA | | EA-D | | | Genome equivalents |
| Patient | Sex | Age | Histopathology | IgA | IgG | IgA | IgG | EBNA | cRNA–DNA |
| K.K.[a] | F | 74 | Undifferentiated | 160 | 2560 | 10 | 1280 | 160 | ND |
| J.J. | M | 50 | Undifferentiated | 10 | 640 | 10 | 20 | 20 | 26 |
| L.A. | F | 44 | Adenocarcinoma | 40 | 320 | < 10 | < 10 | 640 | < 1 |

[a] Biopsy done in Greenland.

ND = not done.

FIG. 4.—Reassociation kinetic analysis on DNA from a case of undifferentiated SGC (J.J.) and adenocarcinoma (L.A.). All samples contained 100 μg/ml cellular DNA and 1 ng of $^{32}$P-EBV DNA. J.J. was determined to contain 17 EBV genome equivalents per cell, while L.A. was clearly negative.

Eskimoes. Furthermore, the serological profiles covered the same range as found elsewhere in patients from high-risk areas (Henle *et al.*, 1970, 1971, 1977; Henle & Henle, 1976; de Thé *et al.*, 1978; Lanier *et al.*, 1981).

Another type of malignancy more common in Eskimoes, as compared to other ethnic groups, is cancer of the salivary glands (Schafer *et al.*, 1975; Nielsen *et al.*, 1978). Over a 20-year period 92% of malignant salivary-gland neoplasms in Greenland were identified as undifferentiated carcinomas (Nielsen *et al.*, 1978). Of the 3 cases of salivary gland carcinomas presented here, 2 had undifferentiated carcinomas and 1 a poorly differentiated adenocarcinoma EBV-specific serum antibody titres indicated an enhanced antigenic stimulation in all 3 cases. That in itself was not surprising since it has long been known that many malignant and non-malignant diseases cause an increase in EBV-specific antibody titres, presumably due to activation of the persistent viral carrier state, which reguarly follows primary infection with EBV (Henle & Henle, 1979*a*). An important finding, however, was the detection of a significant number of EBV genome equivalents in 1 of the 2 biopsy specimens examined, both by cRNA filter hybridization and by vDNA reassociation kinetic analysis. This biopsy specimen, from a parotid gland tumour, contained an undifferentiated carcinoma. This observation corroborates the findings of Lanier *et al.* (1981) of significant numbers of EBV genome equivalents in 2 salivary gland tumours of Alaskan Eskimoes. It has long been suspected that the primary site of infection with EBV is somewhere in the oropharynx, and that EBV may replicate in normal salivary glands, especially the parotid gland (Niederman *et al.*, 1976; Morgan *et al.*, 1979). Furthermore, the parotid gland has been implicated as a possible habitat of EBV by demonstration of EBV genomes in normal gland tissue by *in situ* hybridization and reassociation kinetic analysis (Wolf *et al.*, 1981). Thus the question remains unanswered whether EBV is present merely as a passenger derived from one of its natural sites of persistence, or causally related to undifferentiated salivary-gland carcinoma. In some cases of salivary-gland carcinoma, a secondary NPC cannot be excluded

(Nielsen *et al.*, 1978; Lanier *et al.*, 1981) but, in the 3 cases described here, biopsy samples from the nasopharynx contained no tumour tissue.

EBV is the causative agent of infectious mononucleosis (Henle & Henle, 1979*b*) and is uniquely associated with BL (Epstein & Achong, 1979) and NPC (Anderson-Anvret *et al.*, 1978; Klein, 1979). Recently, a link has also been demonstrated between EBV and certain lymphoproliferative disorders that occur in patients with inherited or acquired immunodeficiencies (Saemundsen *et al.*, 1981). In BL and NPC "association" refers to the regular presence of EBV genomes and the consequent expression of EBNA in all tumour cells. The regular association of a virus with a given tumour, irrespective of geographic differences in its distribution, affords strong evidence for a causal relationship of that virus to that particular cancer. The results presented here thus lend further support to an aetiological role for EBV in NPC. It is clear, however, that because of the unusual geographic and ethnic distribution of NPC, both genetic and environmental factors contribute to the aetiology of this tumour (Anderson-Anvret *et al.*, 1978; Klein, 1979).

We wish to thank Ann-Christine Synnerholm, Ingrid Törnberg, Marie Adams and Sheila Buerkle for their excellent technical assistance.

This work was supported in part by Grant No. 1 R01 CA 3264-01 and Contract No. 1-CP-3-3272 from the National Institute of Health, ACS grant RD-124, King Gustaf V Jubilee Fund and the Swedish Cancer Society.

### REFERENCES

ADAMS, A. (1975) Preparation of Epstein–Barr virus from P3HR-1 cells and isolation of the virus DNA. In *Epstein–Barr Virus Production, Concentration and Purification* (Eds Ablashi *et al.*). Lyon: IARC. p. 126.

ANDERSON-ANVRET, M., FORSBY, N. & KLEIN, G. (1978) Nasopharyngeal carcinoma. *Prog. Exp. Tumor Res.*, **21**, 100.

ANDERSON-ANVRET, M., FORSBY, N., KLEIN, G. & HENLE, W. (1977) Relationship between the Epstein–Barr virus and undifferentiated nasopharyngeal carcinoma: Correlated nucleic acid hybridization and histopathological examination. *Int. J. Cancer.* **20**, 486.

ANDERSON-ANVRET, M., FORSBY, N., KLEIN, G., HENLE, W. & BJÖRKLUND, A. (1979) Relationship between the Epstein–Barr virus genome and nasopharyngeal carcinoma in Caucasian patients. *Int. J. Cancer.* **23**, 762.

CLIFFORD, P. & BEECHER, J. L. (1964) Nasopharyngeal cancer in Kenya: Clinical and environmental aspects. *Br. J. Cancer*, **18**, 25.

DE THÉ, G. (1979) Demographic studies implicating the virus in the causation of Burkitt's lymphoma: Prospects for nasopharyngeal carcinoma. In *The Epstein–Barr Virus* (Eds Epstein & Achong). Berlin: Springer-Verlag. p. 417.

DE THÉ, G., LAVOUE, M. F. & MUENZ, L. (1978) Differences in EBV antibody titres of patients with nasopharyngeal carcinoma originating from high, intermediate and low incidence areas. In *Nasopharyngeal Carcinoma: Etiology and Control* (Eds de Thé & Ito). Lyon: WHO-IARC. p. 471.

EPSTEIN, M. A. & ACHONG, B. G. (1979) The relationship of the virus to Burkitt's lymphoma. In *The Epstein–Barr Virus* (Eds Epstein & Achong). Berlin: Springer-Verlag. p. 322.

EPSTEIN, M. A., ACHONG, B. G. & BARR, Y. M. (1964) Virus particles in cultured lymphoblasts from Burkitt's lymphoma. *Lancet*, i, 702.

EPSTEIN, M. A., HENLE, G., ACHONG, B. G. & BARR, Y. M. (1965) Morphological and biological studies on a virus in cultured lymphoblasts from Burkitt's lymphoma. *J. Exp. Med.*, **121**, 761.

HENLE, G. & HENLE, W. (1976) Epstein–Barr specific IgA serum antibodies as an outstanding feature of nasopharyngeal carcinoma. *Int. J. Cancer*, **17**, 1.

HENLE, W. & HENLE, G. (1979*a*) Seroepidemiology of the virus. In *The Epstein–Barr Virus* (Eds Epstein & Achong). Berlin: Springer-Verlag. p. 61.

HENLE, G. & HENLE, W. (1979*b*) The virus as an etiological agent of infectious mononucleosis. In *The Epstein–Barr Virus* (Eds Epstein & Achong). Berlin: Springer-Verlag. p. 297.

HENLE, W., HENLE, G. & HOROWITZ, C. A. (1974) Epstein–Barr virus-specific diagnostic tests in infectious mononucleosis. *Human Pathol.*, **5**, 551.

HENLE, G., HENLE, W. & KLEIN, G. (1971) Demonstration of two distinct components in the early antigen complex of Epstein–Barr virus infected cells. *Int. J. Cancer*, **8**, 272.

HENLE, W., HENLE, G., HO, H. C. & 7 others (1970) Antibodies to Epstein–Barr virus in nasopharyngeal carcinoma, other head and neck neoplasms and control groups. *J. Natl Cancer Inst.*, **44**, 225.

HENLE, W., HO, J. H. C., HENLE, G., CHAN, J. C. W. & KWAN, H. C. (1977) Nasopharyngeal carcinoma: Significance of changes in Epstein–Barr virus-related antibody patterns. *Int. J. Cancer*, **20**, 663.

HUANG, D. P., HO, J. H. C., HENLE, W. & HENLE, G. (1974) Demonstration of Epstein–Barr virus associated nuclear antigen in nasopharyngeal carcinoma cells from fresh biopsies. *Int. J. Cancer*, **14**, 580.

KLEIN, G. (1979) The relationship of the virus to nasopharyngeal carcinoma. In *The Epstein–Barr Virus* (Eds Epstein & Achong). Berlin: Springer-Verlag. p. 339.

KLEIN, G., GIOVANELLA, B. C., LINDAHL, T., FIALKOW, P. J., SINGH, S. & STEHLIN, J. (1974)

Direct evidence for the presence of Epstein–Barr virus DNA and nuclear antigen in malignant epithelial cells from patients with anaplastic carcinoma of the nasopharynx. *Proc. Natl Acad. Sci.*, **71**, 4737.

Lanier, A. P., Bender, T. R., Blot, W. J., Fraumeni, F. & Hurlbert, W. B. (1976) Cancer incidence in Alaskan natives. *Int. J. Cancer*, **18**, 409.

Lanier, A. P., Bender, T., Talbot, M. & 6 others (1980) Nasopharyngeal carcinoma in Alaskan Eskimoes, Indians and Aleuts: A review of cases and study of Epstein–Barr virus, HLA and environmental risk factors. *Cancer*, **46**, 2100.

Lanier, A. P., Bornkamm, G. W., Henle, W. and 4 others (1981) Association of Epstein–Barr virus with nasopharyngeal carcinoma in Alaskan native patients: Serum antibodies and tissue EBNA and DNA. *Int. J. Cancer*, **28**, 301.

Lindahl, T., Adams, A., Bjursell, G., Bornkamm, G. W., Kaschkădierich, C. & Jehn, U. (1976) Covalently closed circular duplex DNA of Epstein–Barr virus in a human lymphoid cell line. *J. Mol. Biol.*, **102**, 511.

Morgan, D. G., Niederman, J. C., Miller, G., Smith, H. W. & Dowaliby, J. M. (1979) Site of Epstein–Barr virus replication in the orpharynx. *Lancet*, ii, 1154.

Niederman, J. C., Miller, G., Pearson, H. A., Pagano, J. S. & Dowaliby, J. M. (1976) Infectious mononucleosis. Epstein–Barr virus shedding in saliva and the orpharynx. *N. Engl. J. Med.*, **294**, 1355.

Nielsen, N. H., Mikkelsen, F. & Hansen, J. P. H. (1977) Nasopharyngeal cancer in Greenland. The incidence in an arctic Eskimo population. *Acta Path. Microbiol. Scand.* (*Sect. A*), **85**, 850.

Nielsen, N. H., Mikkelsen, F. & Hansen, J. P. H. (1978) Incidence of salivary gland neoplasms in Greenland, with special reference to an anaplastic carcinoma. *Acta Pathol. Microbiol. Scand.* (*Sect. A*), **86**, 185.

Nonoyama, M., Huang, C. H., Pagano, J. S., Klein, G. & Singh, S. (1973) DNA of Epstein–Barr virus detected in tissue of Burkitt's lymphoma and nasopharyngeal carcinoma. *Proc. Natl. Acad. Sci.*, **70**, 3265.

Old, L. J., Boyse, E. A., Ottegen, H. F. and 4 others (1966) Precipitating antibody in human serum to an antigen present in cultured Burkitt's lymphoma cells. *Proc. Natl Acad. Sci.*, **56**, 1699.

Petterson, U. & Sambrook, J. (1973) Amount of viral DNA in the genome of cells transformed by adenovirus type 2. *J. Mol. Biol.*, **73**, 125.

Reedman, G. M. & Klein, G. (1973) Cellular localization of an Epstein–Barr virus (EBV)-associated complement fixing antigen in producer and non-producer lymphoblastoid cell lines. *Int. J. Cancer*, **11**, 499.

Rymo, L. (1979) Identification of transcribed regions of Epstein–Barr virus DNA in Burkitt's lymphoma derived cells. *J. Virol.*, **32**, 8.

Saemundsen, A. K., Purtilo, D. T., Sakamoto, K. & 7 others (1981) Documentation of Epstein–Barr virus infection in immunodeficient patients with life-threatening lymphoproliferative disorders by Epstein–Barr virus complementary RNA/DNA and viral DNA/DNA hybridization. *Cancer Res.*, **41**, 4237.

Schafer, O., Hildes, J. A., Medd, L. M. & Cameron, D. G. (1975) The changing pattern of neoplastic disease in Canadian Eskimoes. *Can. Med. Assoc. J.*, **112**, 1399.

Schmauz, R. & Templeton, A. C. (1972) Nasopharyngeal carcinoma in Uganda. *Cancer*, **29**. 610.

UICC (1979) *TNM Klassifikation der maligne Tumoren*. Berlin: Springer Verlag. p. 25.

Wolf, H., Bayliss, G. J. & Wilmes, E. (1981) Biological properties of Epstein–Barr Virus. In *Cancer Campaign*, Vol. 5, *Nasopharyngeal Carcinoma* (Eds Grundmann *et al.*) Stuttgart: Gustav Fischer Verlag. p. 101.

Wolf, H., zur Hausen, H. & Becker, V. (1973) EB-viral genomes in epithelial nasopharyngeal carcinoma cells. *Nature* (*New Biol*)., **244**, 245.

Wolf, H., zur Hausen, H., Klein, G., Becker, V., Henle, G. & Henle, W. (1975) Attempts to detect virus specific DNA sequences in human tumors. III. Epstein–Barr viral DNA in non-lymphoid nasopharyngeal cells. *Med. Microbiol. Immunol.* **161**, 15.

zur Hausen, H., Schulte-Holthausen, H., Klein, G. & 4 others (1970) EBV DNA in biopsies of Burkitt tumors and anaplastic carcinomas of the nasopharynx. *Nature*, **228**, 1055.

zur Hausen, H., Schulte-Holthausen, H., Wolf, H., Dörries, K. & Egger, H. (1974) Attempts to detect virus-specific DNA in human tumors, II. Nucleic acid hybridization with complementary RNA of human herpes group viruses. *Int. J. Cancer*, **13**, 657.